# Effect of blocking of alpha1-adrenoreceptor isoforms on the noradrenaline-induced changes in contractility of inflamed pig uterus

**DOI:** 10.1371/journal.pone.0280152

**Published:** 2023-02-17

**Authors:** Barbara Jana, Jarosław Całka

**Affiliations:** 1 Division of Reproductive Biology, Institute of Animal Reproduction and Food Research of the Polish Academy of Sciences, Olsztyn, Poland; 2 Department of Clinical Physiology, Faculty of Veterinary Medicine, University of Warmia and Mazury, Olsztyn, Poland; National Dairy Research Institute, INDIA

## Abstract

**Background:**

Disturbances in uterine contractility often lead to the origin, development and maintenance of endometritis and metritis, which are a very common and serious pathologies in domestic animals. Here we aimed to investigate the role of α1A-, α1B- and α1D-adrenoreceptors (ARs) in noradrenaline (NA)-induced contractility of inflammatory-changed porcine uterus.

**Methods:**

On Day 3 of the estrous cycle, either *Escherichia coli* (*E*. *coli*) suspension (*E*. *coli* group) or saline (SAL group) was injected into uterine horns, or only laparotomy was performed (CON group). Eight days later, infected gilts developed severe acute endometritis.

**Results:**

Compared to the period before NA application, NA reduced the contractile amplitude and frequency in myometrium (MYO) and endometrium (ENDO)/MYO strips from the CON, SAL and *E*. *coli* groups. In the last group, the amplitude in MYO and the frequency in ENDO/MYO were lowered versus other groups. After using α1A-ARs antagonist with NA, a greater decrease or occurrence of a drop in the amplitude and frequency in all groups (ENDO/MYO) were found compared to this neurotransmitter action alone. Such results were noted for NA action on the frequency after α1B-ARs blocking in the CON (both kinds of strips) and SAL (ENDO/MYO) groups. In response to α1D-ARs antagonist with NA, a greater decrease or occurrence of a drop in the amplitude was noted in the CON (both kinds of strips) and SAL and *E*. *coli* (MYO) groups. Use of these factors caused the similar changes in the frequency in CON and *E*. *coli* (MYO) and SAL (ENDO/MYO) groups. In response to NA, α1A,B,D-ARs antagonist led to a greater reduction or appearance of a drop in the amplitude in the CON and SAL (ENDO/MYO) and *E*. *coli* (both kinds of strips) as well as in the frequency in the CON and SAL (ENDO/MYO) and *E*. *coli* (MYO) groups.

**Conclusions:**

In conclusion, activation of α1A- and α1D-ARs by NA promotes the contractile amplitude and frequency in the inflamed pig uterus; pharmacological modulation of these receptors can be utilized to enhance systolic activity of myometrium.

## Introduction

The main neurotransmitter of sympathetic component of the nervous system is noradrenaline (NA) which acts by adrenoreceptors (ARs) [1]. ARs are classified into α and β types. α-ARs type includes subtypes: α1 and α2. The α1-ARs have been divided into isoforms, the α1A, α1B and α1D. Isoforms (A, B, C) are distinguished among α2-ARs. The type of β-ARs is divided into subtypes: 1, 2 and 3 [2].

In pig uterus, the most numerous are sympathetic fibers which are found in all layers of the uterus [3, 4]. Under physiological conditions, expression of all isoforms of α1- and α2-ARs, and all subtypes of β-ARs, was revealed in myocytes of myometrium (MYO) in animals [5–13]. Activation of α-ARs by NA stimulates, while activation of β-ARs decreases, myometrial contractility [14–16]. All isoforms of α1-ARs participate in the regulation of rat uterine contractility [5, 6, 8, 17].

Disturbances in the contractility of uterus and immunological processes contribute to the origin, development, and maintenance of endometritis/metritis. These pathologies commonly occur in animals leading to disruption of reproductive processes and reduced profitability of production [18, 19]. Uterine inflammation takes place mainly after parturition, and difficult labour and fetal membrane retention favour their development [20]. Con-tractile activity of uterus under inflammatory conditions depends on the severity and du-ration of inflammation. Increased contractility manifests by discharge of inflammatory exudate from the genital tract, while decreased contractility or its loss lead to the accumulation of mucopurulent exudate in the uterus [18, 19, 21].

In relation to sympathetic innervation of inflamed uterus, it is only known that *Escherichia coli* (*E*. *coli*)-induced endometritis in pig leads to alterations in the neurochemical characteristics of uterine neurons in caudal mesenteric ganglion [22] and paracervical ganglion [23], including a rise in noradrenergic neurons population. In the uteri of rats with general inflammation, a drop in the NA release from nerve fibers and in β-AR-mediated relaxation, rise in α-AR-mediated contraction, as well as changes in ARs expression (including α1-ARs isoforms) were found [10]. In inflamed pig uterus, presence of all α1- and α2-ARs isoforms and all β-ARs subtypes [13], and the lowering effect of NA on its contractility were also revealed [24]. Moreover, the role of particular β-ARs subtypes [16] and particular α2-ARs isoforms [25] in the contractility of pig uterus with inflammation was documented. No data are available on the role of particular α1-ARs isoforms in the contractility of inflamed uterus. Dysregulation of myometrial contractile response by adrenergic stimuli influencing via particular isoforms of α1-ARs can be significant for the course and/or outcome of inflammation. It is therefore crucial to understand more deeply the receptor mechanism regulating NA effect on inflamed uterus. The obtained results will be able to contribute to the improvement of prophylaxis and treatment of uterine inflammation in animals.

Here, we performed contractile tension, amplitude, and frequency measurements in the inflamed porcine uterus to investigate the participation of α1A-, α1B- and α1D-ARs isoforms in NA-induced changes.

## Materials and methods

### Animals

Local Ethics Committee for Experiments on Animals (University of Warmia and Mazury in Olsztyn, Poland) approved all study procedures (Consent no. 65/2015). The guidelines in EU Directive 2010/63/EU for animals experiments were taken into account. Research was carried out on 15 gilts (Large White x Landrace, age 7–8 months, body weight 107.3 ± 1.8 kg /mean ± SEM/). A tester boar detected behavioural estrus. Gilts chosen for the study did not exhibit reproductive disorders: discharges from vagina were not observed and the second estrous cycle took place regularly. The animals were transported from a commercial farm (Agro-Wronie Sp. z o. o., Wronie, Wąbrzeźno, Poland) to the local animal house (University of Warmia and Mazury, Olsztyn, Poland) three days prior to testing (acclimatization period). They stayed in individual pens (an area of about 5 m^2^), at a temperature of 18±2°C and the following light conditions: natural daylight—14.5±1.5 h, night—9.5±1.5 h. Gilts received commercial diet and had access to water.

### Study design

After the acclimatization period, the gilts were assigned randomly (day 3 of the second estrous cycle—day 0 of the research), into following groups (n = 5 in each group): *Escherichia coli* (*E*. *coli*) group (gilts with intrauterine *E*. *coli* injections), SAL group (gilts with intrauterine saline injections), and CON group (gilts with a "sham" operation only).

Experiment procedures have been published earlier [24]. Most importantly, following premedication (by atropine, azaperone, and ketamine hydrochloride) and general anaesthesia (by ketamine hydrochloride) median laparotomy was performed. 50 ml of *E*. *coli* suspension (content: 109 cfu/ml, bacterial strain O25:K23/a/:H1; Department of Microbiology, National Veterinary Research Institute, Puławy, Poland) were injected into each uterine horn in the *E*. *coli* group. In turn, in the SAL group, 50 ml of saline solution were injected. Bacterial suspension and saline solution were injected into each horn in five places (10 ml/each place) at a similar distance from each other. After that, horns were massaged to evenly place *E*. *coli* suspension and saline. In the animals of CON group, only median laparotomy was performed. All study gilts were untreated in the time from surgery through euthanasia. Eight days later (expected day 11 of the estrous cycle), the pigs were euthanised (by an overdose of sodium pentobarbital) and their uteri were collected. For study of the uterine contractile activity, fragments of the horns collected from their middle parts were transported (on ice) to the laboratory within 5 min of collection.

### Separation of uterine strips and recordings isometric contractile activity

In pigs, the participation of prostaglandins (PGs), leukotrienes (LTs), NA and acetylcholine (ACh) on contractile parameters (amplitude, frequency, as well as tension) partly differs between MYO and endometrium (ENDO)/MYO strips [24, 26–29]. Similarly, in the present research, two types of strips (MYO, ENDO/MYO) were collected. The strips (approximate size 3 x 5 mm) were rinsed in saline and mounted between two stainless steel hooks in an organ bath with capacity of 10 ml (Radnoti Unit Tissue Organ Bath System type 159920, Germany) under 5 mN tension. Uterine strips were kept in Krebs-Ringer solution (in mM/l: NaCl, 120.3; KCl, 5.9; CaCl2, 2.5; MCl2, 1.2; NaHCO3, 15.5; glucose, 11.5; pH 7,4), at a temperature of 37°C, which was constantly oxygenated with gas mixture of 95% O2 and 5% CO2.

**Fig 1:**
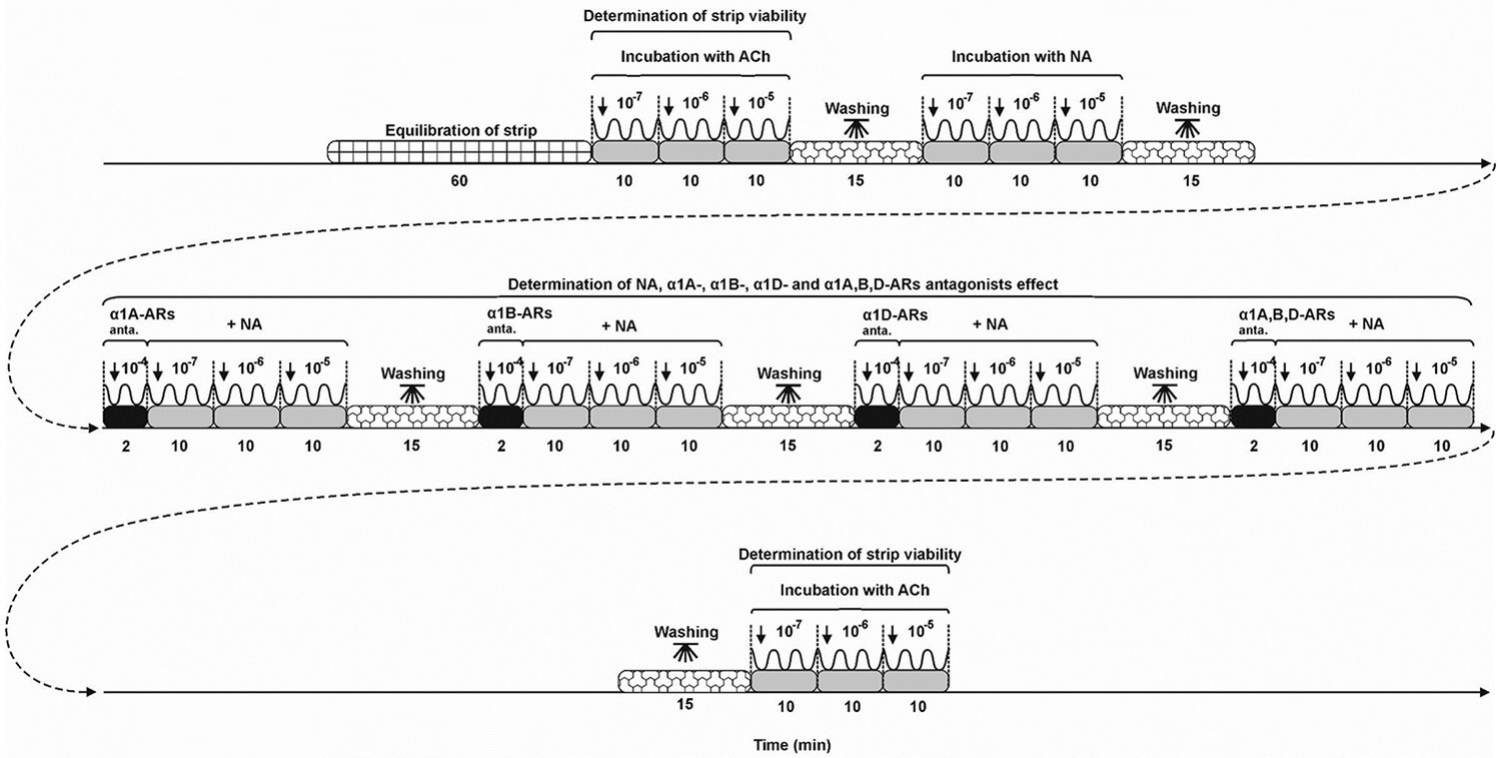
Diagram showing treatment of uterine strips. ACh–acetylcholine; NA—noradrenaline; α1A-ARs anta.–antagonist of isoform A of adrenoreceptor subtype α1; α1B-ARs anta.–antagonist of isoform B of adrenoreceptor subtype α1; α1D-ARs anta.—antagonist of isoform D of adrenoreceptor subtype α1; α1A,B,D-ARs anta.–antagonist of isoforms A,B,D of adrenoreceptor subtype α1. Substance concentrations are given in moles.

The tissue tension (resting/baseline tension expressed in mN), amplitude (difference between the minimum and maximum value for a single contraction /in mN/), and frequency (number of peaks) of contractions were measured by a force displacement transducer and recorded and analyzed on a computer using PowerChart software (Chart v5, scope v5, AD Instruments). Fig 1 illustrates the measurement of uterine contractility.

Before the isometric contractile recordings, the strips were allowed to equilibrate for at least 60 min in the oxygenated Krebs solution at 37°C. Then, to determine the contractile functionality of strips, they were exposed to ACh (Sigma, St. Louis, MO, USA) at doses of 10–7, 10–6, and 10–5 M. ACh uterine action was reported previously [29]. Afterwards, strips were exposed to NA (Levonor, Warszawskie Zakłady Farmaceutyczne Polfa, Poland) at doses of 10^−7^, 10^−6^, and 10^−5^ M. The ACh and NA effects at each dose on the contractility were registered for 10 min. Results of NA influence on uterine contractility were published previously [16]. Values of parameters under the NA influence were also recorded after the use of following antagonists for: α1A- (RS 17053 hydrochloride, cat. no. 0985), α1B- (Rec 15/2615 dihydrochloride, cat. no. 3284), α1D- (BMY 7378 dihydrochloride, cat. no. 1006) and α1A,B,D- (Doxazosin mesylate, cat. no. 2964) ARs, all from Tocris Bioscience. First, the uterine strips were under the action of α1A-, α1B-, α1D- and α1A,B,D-ARs antagonists (dose: 10–4 M) for 2 min, and then NA (doses: 10^−7^, 10^−6^, 10^−5^ M) was administered. The effects of particular antagonist and NA on uterine contractility were registered for 10 min. After each treatment, the tissues were washed three times in Krebs-Ringer solution. At the end of the measurement session, the strips were re-treated with ACh (at doses as above) to assess their contractile activity. Doses of ACh and NA were given in the reports [26, 27] and the efficiency of α1A-, α1B-,α1D- and α1A,B,D-ARs antagonists doses were established in preliminary experiment (data not shown).

### Statistical analyses

Only the results for which the variability (parameters obtained in response to ACh between the beginning and the end of investigation) was below 20% were statistically analyzed. Tension, amplitude and frequency of the uterine strips before the addition of the substances (pre-treatment period) and following their use (ACh at each dose /10^−7^, 10^−6^, 10^−5^ M/; NA at each dose /10^−7^, 10^−6^, 10^−5^ M/; NA at each dose /10^−7^, 10^−6^, 10^−5^ M/ with α1A-, α1B-, α1D- and α1A,B,D-ARs antagonists /a dose of 10^−4^ M/) were counted for 10 min. Values of parameters (mean ± SEM) counted for each group before the addition of the substances were established as 100%. Influences of substances at each dose were expressed as a per-centage (mean ± SEM) of value before their addition. Statistical analyses included com-parisons between 1) mean values before and after each treatment (NA alone, NA and α1A-, α1B-, α1D-, α1A,B,D-ARs antagonists) in each group, 2) mean values between groups in response to the same treatment (NA alone, NA and α1A-, α1B-, α1D-, α1A,B,D-ARs antagonists), 3) mean values between treatments (NA and α1A-, α1B-, α1D-, α1A,B,D-ARs antagonists versus NA alone) for each group/NA dose. Statistical significance of the obtained data was assessed by a Bonferroni test (two-way ANOVA, InStat Graph Pad, San Diego, CA, USA). Statistically significant differences were indicated by: *P<0.05, **P<0.01, ***P<0.001. All obtained data were normally distributed. No statistical power calculation was conducted prior to the research, and the establishment of the size of experimental groups was based on previous studies, where the number of five animals in the case of pigs during uterine investigations is commonly accepted.

## Results

### Effect of NA alone or with α1-ARs isoform antagonists on the contractile tension of MYO

#### Comparison of NA effect in particular groups to the period before its addition

**Fig 2:**
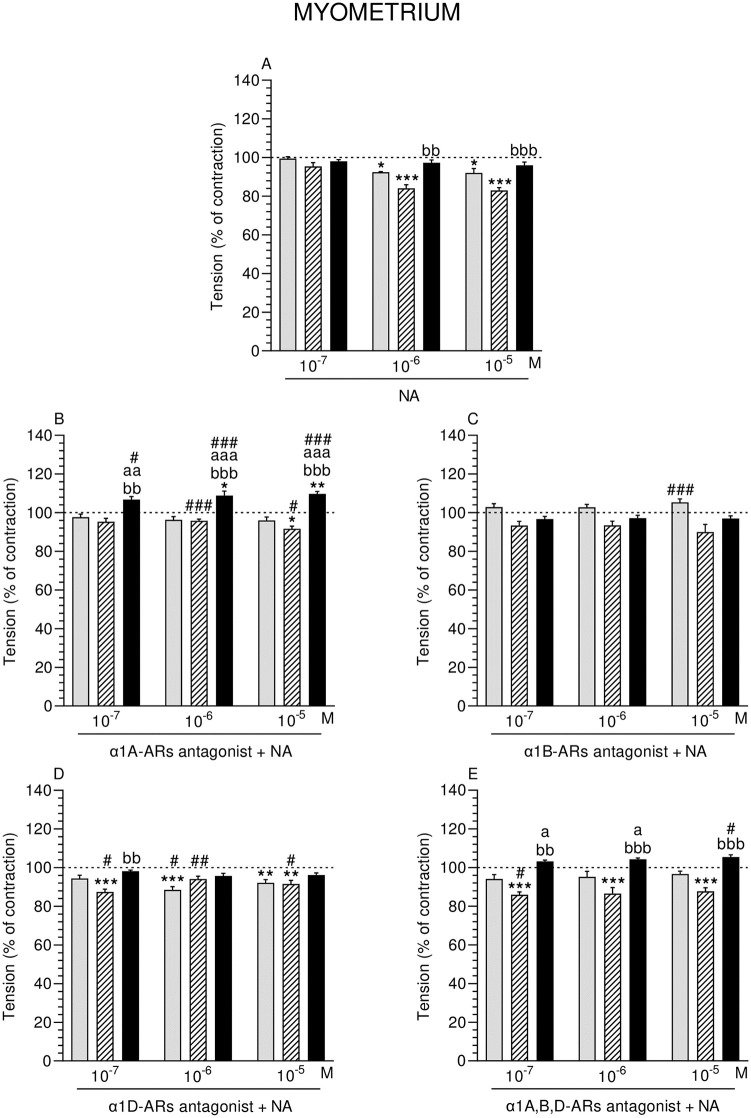
Effect of noradrenaline (NA) alone (A) and NA with α1A- (B), α1B- (C), α1D- (D) and α1A,B,D (E)–adrenoreceptors (ARs) antagonists (a dose of 10^−4^ M) on the tension of con-tractions in pig myometrium strips obtained from the CON (grey bars), SAL (hatched bars), and *E*. *coli* (black bars) groups. Data from five experiments (gilts, in each group). Effects of particular doses of NA and antagonists are expressed as percentage (mean ± SEM) in relation to the basal (pre-treatment period) contractile tension, established as 100% (horizontal lines). *P<0.05, **P<0.01, ***P<0.001—indicate differences from baseline in each group; ^a^P<0.05, ^aa^P<0.01, ^aaa^P<0.001- indicate differences between CON and *E*. *coli* groups for the same treatment; ^bb^P<0.01, ^bbb^P<0.001—indicate differences between SAL and *E*. *coli* groups for the same treatment; ^#^P<0.05, ^##^P<0.01, ^###^P<0.001 compared between particular antagonist with NA effect versus NA effect alone for the same group/NA dose.

MYO tension in the CON and SAL groups was significantly dropped by NA (10^−6^, 10^−5^ M) (Fig 2A).

#### Comparison of NA effect between groups

Tension in MYO of the *E*. *coli* group was significantly higher than in the SAL group after using NA (10^−6^, 10^−5^ M) (Fig 2A).

#### Comparison of α1-ARs isoform antagonists and NA effect in particular groups to the period before their addition

α1A-ARs antagonist and NA significantly dropped the tension in MYO of the SAL group (NA: 10^−5^ M), and significantly elevated it in the *E*. *coli* group (NA: 10^−6^, 10^−5^ M) (Fig 2B). α1D-ARs antagonist and NA significantly reduced the tension in the CON (NA: 10^−6^, 10^−5^ M) and SAL (NA: 10^−7^, 10^−5^ M) groups (Fig 2D). In the SAL group, α1A,B,D-ARs antagonist and NA (three doses) significantly decreased the tension (Fig 2E).

#### Comparison of α1-ARs isoform antagonists and NA effect between groups

Tension in MYO of the *E*. *coli* group was significantly higher than in other groups after using α1A-ARs antagonist and NA (three doses) (Fig 2B). This parameter was significantly in-creased in the *E*. *coli* group by α1D-ARs antagonist and NA (10^−7^ M) versus the SAL group (Fig 2D). α1A,B,D-ARs antagonist and NA significantly raised the tension in MYO of the *E*. *coli* group compared to the CON (NA: 10^−7^, 10^−6^ M) and SAL (NA: three doses) groups (Fig 2E).

#### Comparison of α1-ARs isoform antagonists and NA effect to NA effect alone

α1A-ARs antagonist and NA significantly increased the tension in MYO of the SAL (NA: 10^−6^, 10^−5^ M) and *E*. *coli* (NA: three doses) groups (Fig 2A and 2B). Similar reaction was evoked by α1B-ARs antagonist and NA (10–5 M) in the CON group (Fig 2A and 2C). α1D-ARs antagonist and NA significantly reduced the tension in the CON (NA: 10^−6^ M) and SAL (NA: 10^−7^ M) groups, and significantly elevated it in the SAL group (NA: 10^−6^, 10^−5^ M) (Fig 2A and 2D). α1A,B,D-ARs antagonist and NA significantly reduced the tension in the SAL group (NA: 10^−7^ M) and significantly elevated it in the *E*. *coli* group (NA: 10^−5^ M) (Fig 2A and 2E).

### Effect of NA alone or with α1-ARs isoform antagonists on the contractile tension of ENDO/MYO

#### Comparison of NA effect in particular groups to the period before its addition

**Fig 3:**
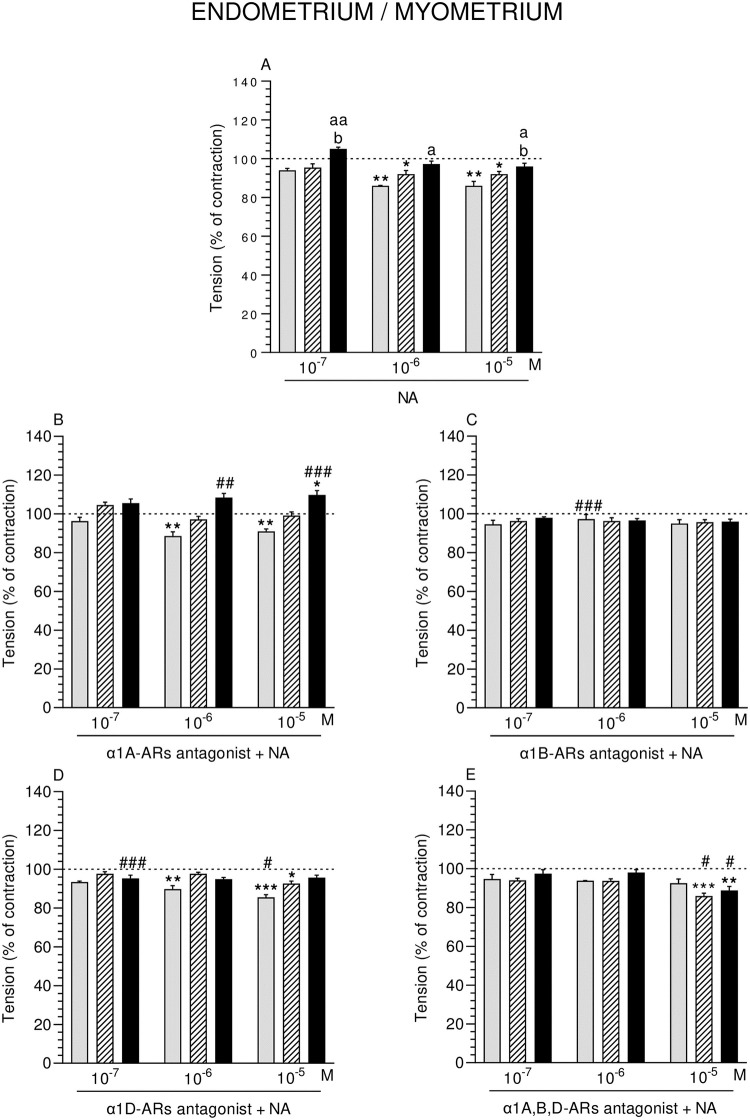
Effect of noradrenaline (NA) alone (A) and NA with α1A- (B), α1B- (C), α1D- (D) and α1A,B,D (E)–adrenoreceptors (ARs) antagonists (a dose of 10^−4^ M) on the tension of con-tractions in pig endometrium/myometrium strips obtained from the CON (grey bars), SAL (hatched bars), and *E*. *coli* (black bars) groups. Data from five experiments (gilts, in each group). Effects of particular doses of NA and antagonists are expressed as percentage (mean ± SEM) in relation to the basal (pre-treatment period) contractile tension, established as 100% (horizontal lines). *P<0.05, **P<0.01, ***P<0.001—indicate differences from baseline in each group; ^a^P<0.05, ^aa^P<0.01- indicate differences between CON and *E*. *coli* groups for the same treatment; ^b^P<0.05—indicates differences between SAL and *E*. *coli* groups for the same treatment; ^#^P<0.05, ^##^P<0.01, ^###^P<0.001 compared between particular antagonist with NA effect versus NA effect alone for the same group/NA dose.

EN-DO/MYO tension in the CON and SAL groups was significantly reduced by NA (10^−6^, 10^−5^ M) (Fig 3A).

#### Comparison of NA effect between groups

In ENDO/MYO of the *E*. *coli* group, NA significantly enhanced the tension versus the CON (NA: three doses) and SAL (NA: 10^−7^, 10^−5^ M) groups (Fig 3A).

#### Comparison of α1-ARs isoform antagonists and NA effect in particular groups to the period before their addition

ENDO/MYO tension in the CON group was significantly reduced by α1A-ARs antagonist and NA (10^−6^, 10^−5^ M), while these factors (NA: 10^−5^ M) significantly increased the tension in the *E*. *coli* group (Fig 3B). α1D-ARs antagonist and NA decreased the tension in ENDO/MYO of the CON (NA: 10^−6^, 10^−5^ M) and SAL (NA: 10^−5^ M) groups (Fig 3D). Tension in the SAL and *E*. *coli* groups was reduced by α1A,B,D-ARs antagonist and NA (10^−5^ M) (Fig 3E).

#### Comparison of α1-ARs isoform antagonists and NA effect between groups

Tension in ENDO/MYO did not differ significantly between groups after using α1A- (Fig 3B), α1B- (Fig 3C), α1D- (Fig 3D) and α1A,B,D (Fig 3E) -ARs antagonists and NA (three doses).

#### Comparison of α1-ARs isoform antagonists and NA effect to NA effect alone

Tension was significantly increased in ENDO/MYO of the *E*. *coli* group by α1A-ARs antagonist and NA (10^−6^, 10^−5^ M) (Fig 2A and 2B), and in the CON group by α1B-ARs antagonist and NA (10^−6^ M) (Fig 3A and 3C). α1D-ARs antagonist and NA reduced the tension in the CON (10^−5^ M) and *E*. *coli* (10^−7^ M) groups (Fig 3A and 3D). Similar influence was exerted by α1A,B,D-ARs antagonist and NA (10^−5^ M) in the SAL and *E*. *coli* groups (Fig 3A and 3E).

### Effect of NA alone or with α1-ARs isoform antagonists on the contractile amplitude of MYO

#### Comparison of NA effect in particular groups to the period before its addition

**Fig 4:**
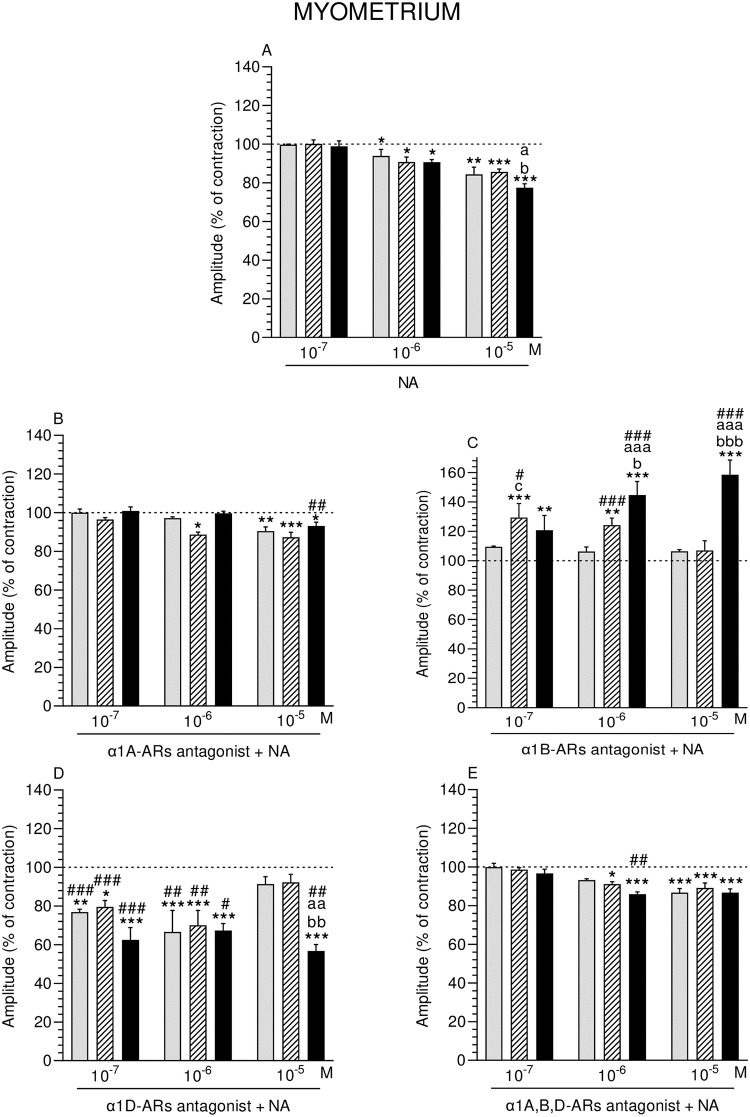
Effect of noradrenaline (NA) alone (A) and NA with α1A- (B), α1B- (C), α1D- (D) and α1A,B,D (E)–adrenoreceptors (ARs) antagonists (a dose of 10^−4^ M) on the amplitude of contractions in pig myometrium strips obtained from the CON (grey bars), SAL (hatched bars), and *E*. *coli* (black bars) groups. Data from five experiments (gilts, in each group). Effects of particular doses of NA and antagonists are expressed as percentage (mean ± SEM) in relation to the basal (pre-treatment period) contractile amplitude, established as 100% (horizontal lines). *P<0.05, **P<0.01, ***P<0.001—indicate differences from baseline in each group; ^a^P<0.05, ^aa^P<0.01, ^aaa^P<0.001- indicate differences between CON and *E*. *coli* groups for the same treatment; ^b^P<0.05, ^bb^P<0.01, ^bbb^P<0.001—indicate differences between SAL and *E*. *coli* groups for the same treatment; ^#^P<0.05, ^##^P<0.01, ^###^P<0.001 compared between particular antagonist with NA effect versus NA effect alone for the same group/NA dose.

Amplitude in MYO of all groups was significantly dropped by NA (10^−6^, 10^−5^ M) (Fig 4A).

#### Comparison of NA effect between groups

Amplitude in MYO of the *E*. *coli* group was significantly lower in response to NA (10^−5^ M) versus other groups (Fig 4A).

#### Comparison of α1-ARs isoform antagonists and NA effect in particular groups to the period before their addition

α1A-ARs antagonist and NA significantly dropped the amplitude in MYO of the SAL group (NA: 10^−6^ M) and of all groups (NA: 10^−5^ M). (Fig 4B). α1B-ARs antagonist and NA significantly increased the amplitude in MYO of the SAL (NA: 10^−7^, 10^−6^ M) and *E*. *coli* (NA: three doses) groups (Fig 4C). In the CON and SAL groups, α1D-ARs antagonist and NA (10^−7^, 10^−6^ M) significantly decreased the amplitude, while in the *E*. *coli* group it was exerted by the antagonist and NA (three doses) (Fig 4D). α1A,B,D-ARs antagonist and NA significantly reduced the amplitude in MYO of the SAL and *E*. *coli* groups (NA: 10^−6^ M) and in all groups (NA: 10^−5^ M) (Fig 4E).

#### Comparison of α1-ARs isoform antagonists and NA effect between groups

After addition of α1B-ARs antagonist and NA, the amplitude in MYO of the SAL group was significantly higher versus the CON group (NA: 10^−7^ M), and in the *E*. *coli* group than in other groups (NA: 10^−6^, 10^−5^ M) (Fig 4C). After using α1D-ARs antagonist and NA (10^−5^ M), the amplitude in MYO of the *E*. *coli* group was significantly lower compared to other groups (Fig 4D).

#### Comparison of α1-ARs isoform antagonists and NA effect to NA effect alone

α1A-ARs antagonist and NA (10^−5^ M) significantly increased the amplitude in MYO of the *E*. *coli* group (Fig 4A and 4B). Similar reaction was evoked by α1B-ARs antagonist and NA in the SAL (NA: 10^−7^, 10^−6^ M) and *E*. *coli* (NA: 10^−6^, 10^−5^ M) groups (Fig 4A and 4C). α1D-ARs antagonist and NA significantly dropped the amplitude in MYO of the CON, SAL (NA: 10^−7^, 10^−6^ M) and *E*. *coli* (NA: three doses) groups (Fig 4A and 4D). α1A,B,D-ARs antagonist and NA (10^−6^ M) significantly reduced it in the *E*. *coli* group (Fig 4A and 4E).

### Effect of NA alone or with α1-ARs isoform antagonists on the contractile amplitude of ENDO/MYO

#### Comparison of NA effect in particular groups to the period before its addition

**Fig 5:**
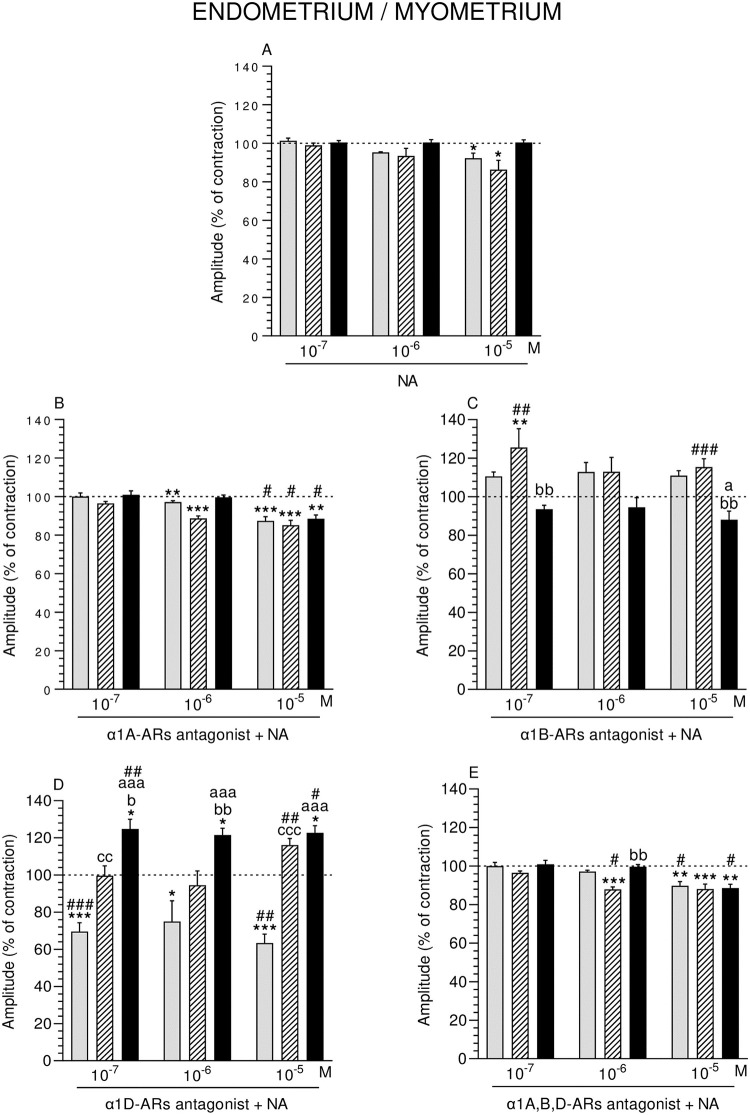
Effect of noradrenaline (NA) alone (A) and NA with α1A- (B), α1B- (C), α1D- (D) and α1A,B,D (E)–adrenoreceptors (ARs) antagonists (a dose of 10^−4^ M) on the amplitude of contractions in pig endometrium/myometrium strips obtained from the CON (grey bars), SAL (hatched bars), and *E*. *coli* (black bars) groups. Data from five experiments (gilts, in each group). Effects of particular doses of NA and antagonists are expressed as percentage (mean ± SEM) in relation to the basal (pre-treatment period) contractile amplitude, established as 100% (horizontal lines). *P<0.05, **P<0.01, ***P<0.001—indicate differences from baseline in each group; ^a^P<0.05, ^aaa^P<0.001- indicate differences between CON and *E*. *coli* groups for the same treatment; ^b^P<0.05, _bb_P<0.01, ^bbb^P<0.001—indicate differences between SAL and *E*. *coli* groups for the same treatment; ^cc^P<0.01, ^ccc^P<0.001—indicate differences between CON and SAL groups for the same treatment; ^#^P<0.05, ^##^P<0.01, ^###^P<0.001 compared between particular antagonist with NA effect versus NA effect alone for the same group/NA dose.

In ENDO/MYO amplitude of the CON and SAL groups, NA (10^−5^ M) significantly reduced (Fig 5A).

#### Comparison of NA effect between groups

In response to NA (three doses) the amplitude in ENDO/MYO did not differ significantly between groups (Fig 5A).

#### Comparison of α1-ARs isoform antagonists and NA effect in particular groups to the period before their addition

After using α1A-ARs antagonist and NA, the amplitude in ENDO/MYO of the CON and SAL groups (NA:10^−6^ M) and in all groups (NA: 10^−5^ M) was significantly reduced (Fig 5B). α1B-ARs antagonist and NA (10^−7^ M) significantly in-creased the amplitude in the SAL group (Fig 5C). α1D-ARs antagonist and NA (three doses) significantly decreased the amplitude in ENDO/MYO of the CON group, while significantly enhanced it in the *E*. *coli* group (Fig 5D). α1A,B,D-ARs antagonist and NA significantly dropped the amplitude in the SAL group (NA: 10^−6^ M) and in all groups (NA: 10^−5^ M) (Fig 5E).

#### Comparison of α1-ARs isoform antagonists and NA effect between groups

After addition of α1B-ARs antagonist and NA, the amplitude in ENDO/MYO of the *E*. *coli* group was significantly lower than in the CON (NA: 10^−5^ M) and SAL (NA: 10^−7^, 10^−5^ M) groups (Fig 5C). This parameter in the SAL group was significantly raised by α1D-ARs antagonist and NA (10^−7^, 10^−5^ M) versus the CON group (Fig 5D). Similar reaction was found between the *E*. *coli* and CON (NA: three doses) and SAL (NA: 10^−7^, 10^−6^ M) groups. α1A,B,D-ARs antagonist and NA (10^−6^ M) significantly elevated the amplitude in the *E*. *coli* group versus the SAL group (Fig 5E).

#### Comparison of α1-ARs isoform antagonists and NA effect to NA effect alone

After using α1A-ARs antagonist and NA (10^−5^ M), the amplitude in ENDO/MYO of all groups significantly lowered (Fig 5A and 5B). A significant rise was found in MYO of the SAL group in response to α1B-ARs antagonist and NA (10^−7^, 10^−5^ M) (Fig 5A and 5C). In response to α1D-ARs antagonist and NA, amplitude in the CON group (NA: 10^−7^, 10^−5^ M) was significantly lowered, while this parameter was significantly increased in the SAL (NA: 10^−5^ M) and *E*. *coli* (NA: 10^−7^, 10^−5^ M) groups (Fig 5A and 5D). α1A,B,D-ARs antagonist and NA significantly decreased the amplitude in the CON and *E*. *coli* (NA: 10^−5^ M) and SAL (NA: 10^−6^ M) groups (Fig 5A and 5E).

### Effect of NA alone or with α1-ARs isoform antagonists on the contractile frequency of MYO

#### Comparison of NA effect in particular groups to the period before its addition

**Fig 6:**
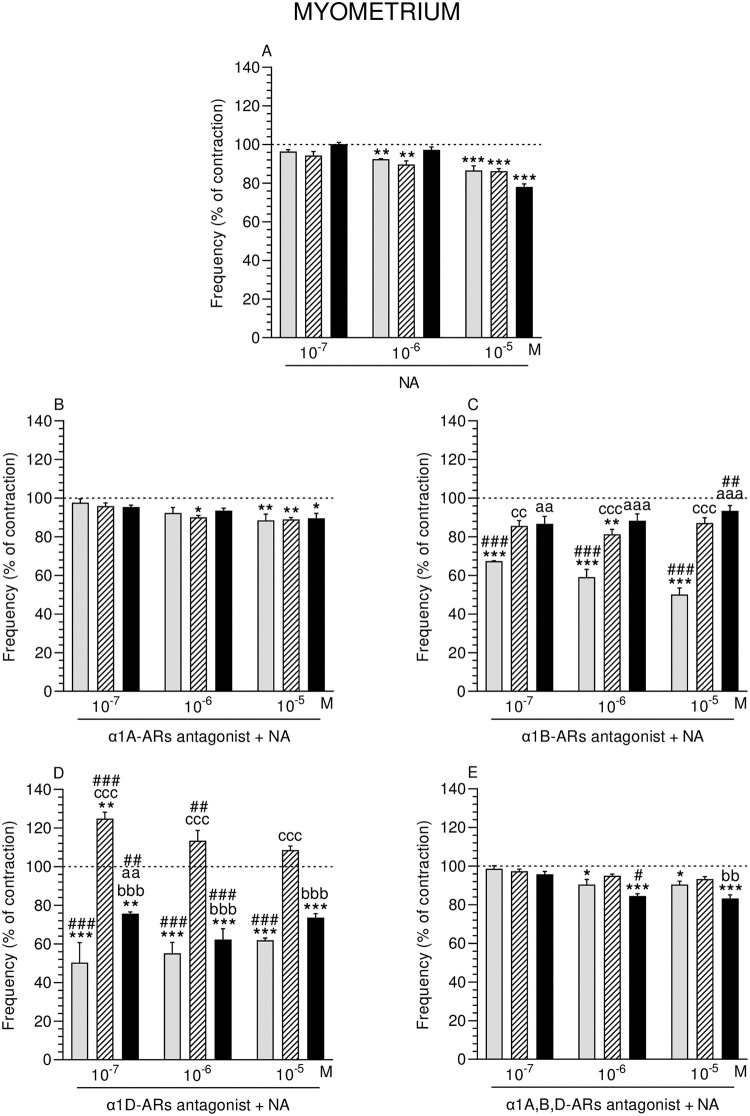
Effect of noradrenaline (NA) alone (A) and NA with α1A- (B), α1B- (C), α1D- (D) and α1A,B,D (E)–adrenoreceptors (ARs) antagonists (a dose of 10^−4^ M) on the frequency of contractions in pig myometrium strips obtained from the CON (grey bars), SAL (hatched bars), and *E*. *coli* (black bars) groups. Data from five experiments (gilts, in each group). Effects of particular doses of NA and antagonists are expressed as percentage (mean ± SEM) in relation to the basal (pre-treatment period) contractile frequency, established as 100% (horizontal lines). *P<0.05, **P<0.01, ***P<0.001—indicate differences from baseline in each group; ^aa^P<0.01, ^aaa^P<0.001- indicate differences between CON and *E*. *coli* groups for the same treatment; ^bb^P<0.01, ^bbb^P<0.001—indicate differences between SAL and *E*. *coli* groups for the same treatment; ^cc^P<0.01, ^ccc^P<0.001—indicate differences between CON and SAL groups for the same treatment; ^#^P<0.05, ^##^P<0.01, ^###^P<0.001 compared between particular antagonist with NA effect versus NA effect alone for the same group/NA dose.

Noradrenaline (10^−6^ M) significantly reduced the frequency in MYO of the CON and SAL groups and in all groups (NA: 10^−5^ M) (Fig 6A).

#### Comparison of NA effect between groups

MYO frequency did not significantly differ between groups after application of NA (three doses) (Fig 6A).

#### Comparison of α1-ARs isoform antagonists and NA effect in particular groups to the period before their addition

α1A-ARs antagonist and NA significantly dropped the frequency in MYO of the SAL group (NA: 10^−6^ M) and in all groups (NA: 10^−5^ M) (Fig 6B). α1B-ARs antagonist and NA significantly lowered the frequency in the CON (NA: three doses) and SAL (NA: 10^−6^ M) groups (Fig 6C). α1D-ARs antagonist and NA (three doses) significantly decreased the frequency in the CON and *E*. *coli* groups, and significantly increased it in the SAL group (NA: 10^−7^ M) (Fig 6D). Frequency in the CON and *E*. *coli* groups was significantly lowered by α1A,B,D-ARs antagonist and NA (10^−6^, 10^−5^ M) (Fig 6E).

#### Comparison of α1-ARs isoform antagonists and NA effect between groups

Frequency in MYO of the SAL and *E*. *coli* groups was significantly raised by α1B-ARs antagonist and NA (three doses) versus the CON group (Fig 6C). Frequency in the SAL group was significantly increased by α1D-ARs antagonist and NA (three doses) compared to the CON and *E*. *coli* groups (Fig 6D). This parameter in the *E*. *coli* group was significantly increased by the antagonist and NA (10^−7^ M) versus the CON group. After using α1A,B,D-ARs antagonist and NA (10^−5^ M), frequency in the *E*. *coli* group was significantly lower compared to the SAL group (Fig 6E).

#### Comparison of α1-ARs isoform antagonists and NA effect to NA effect alone

α1B-ARs antagonist with NA significantly dropped the MYO frequency in the CON group (NA: three doses) and increased it in the *E*. *coli* group (NA: 10^−5^ M) (Fig 6A and 6C). Frequency in the CON group was significantly lowered by α1D-ARs antagonist and NA (three doses), and in the *E*. *coli* group by the antagonist and NA (10^−7^, 10^−6^ M) (Fig 6A and 6D). In the SAL group, these factors (NA: 10^−7^, 10^−6^ M) significantly raised this parameter. Frequency in MYO of the *E*. *coli* group was significantly dropped by α1A,B,D-ARs antagonist and NA (10^−6^ M) (Fig 6A and 6E).

### Effect of NA alone or with α1-ARs isoform antagonists on the contractile frequency of ENDO/MYO

#### Comparison of NA effect in particular groups to the period before its addition

**Fig 7:**
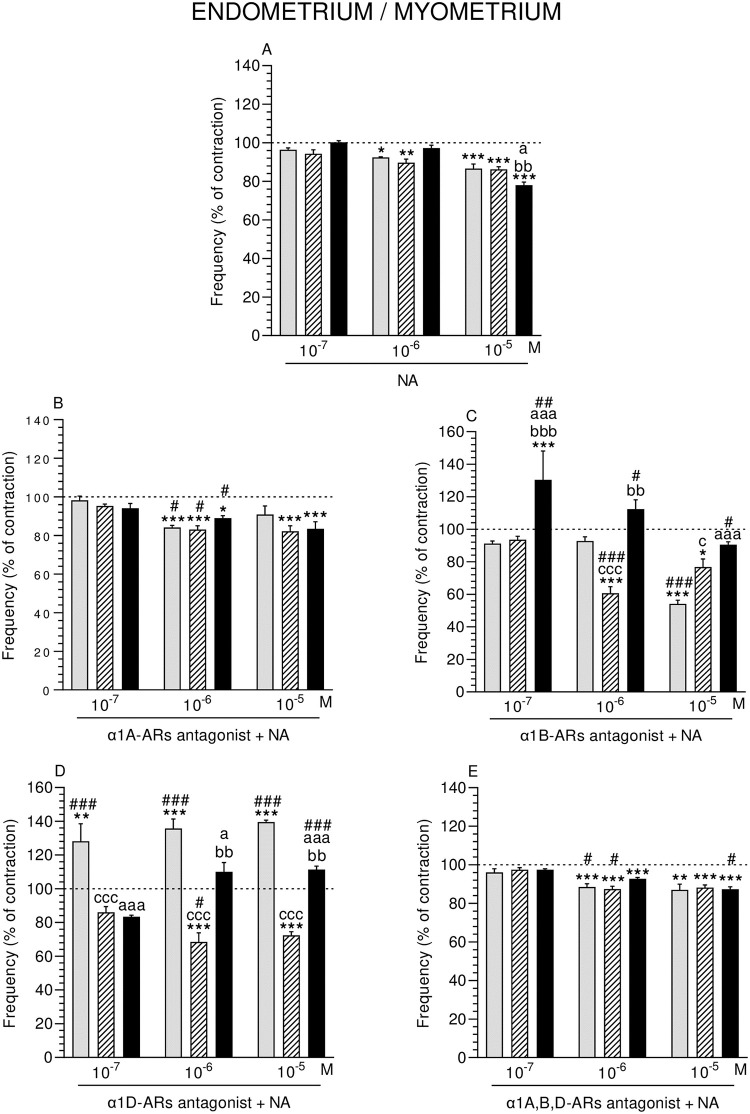
Effect of noradrenaline (NA) alone (A) and NA with α1A- (B), α1B- (C), α1D- (D) and α1A,B,D (E)–adrenoreceptors (ARs) antagonists (a dose of 10^−4^ M) on the frequency of contractions in pig endometrium/myometrium strips obtained from the CON (grey bars), SAL (hatched bars), and *E*. *coli* (black bars) groups. Data from five experiments (gilts, in each group). Effects of particular doses of NA and antagonists are expressed as percentage (mean ± SEM) in relation to the basal (pre-treatment period) contractile frequency, established as 100% (horizontal lines). *P<0.05, **P<0.01, ***P<0.001—indicate differences from baseline in each group; ^a^P<0.05, ^aaa^P<0.001- indicate differences between CON and *E*. *coli* groups for the same treatment; ^bb^P<0.01, ^bbb^P<0.001—indicate differences between SAL and *E*. *coli* groups for the same treatment; ^c^P<0.05, ^ccc^P<0.001—indicate differences between CON and SAL groups for the same treatment; ^#^P<0.05, ^##^P<0.01, ^###^P<0.001 compared between particular antagonist with NA effect versus NA effect alone for the same group/NA dose.

A significant drop in the frequency of ENDO/MYO of the CON and SAL groups was exerted by NA (10^−6^ M), and in all groups (NA: 10^−5^ M) (Fig 7A).

#### Comparison of NA effect between groups

After using NA (10^−5^ M), frequency in ENDO/MYO of the *E*. *coli* group was significantly lower than in other groups (Fig 7A).

#### Comparison of α1-ARs isoform antagonists and NA effect in particular groups to the period before their addition

α1A-ARs antagonist and NA significantly decreased the frequency in ENDO/MYO of the CON (NA: 10^−6^ M), SAL and *E*. *coli* (NA: 10^−6^, 10^−5^ M) groups (Fig 7B). This parameter in the CON (NA: 10^−5^ M) and SAL (NA: 10^−6^, 10^−5^ M) groups was significantly reduced by α1B-ARs antagonist and NA, while in the *E*. *coli* group it was significantly raised by these factors (NA: 10^−7^ M) (Fig 7C). α1D-ARs antagonist and NA significantly increased the frequency in ENDO/MYO of the CON group (NA: three doses), and significantly dropped it in the SAL group (NA: 10^−6^, 10^−5^ M) (Fig 7D). α1A,B,D-ARs antagonist and NA (10^−6^, 10^−5^ M), significantly reduced the frequency in all groups (Fig 7E).

#### Comparison of α1-ARs isoform antagonists and NA effect between groups

Compared to the CON group, frequency in ENDO/MYO of the SAL group was significantly dropped by α1B-ARs antagonist and NA (10^−6^ M), while it was significantly increased by these factors (NA: 10^−5^ M) (Fig 7C). In the *E*. *coli* group, frequency was significantly elevated by this antagonist and NA versus the CON (NA: 10^−7^, 10^−5^ M) and SAL (NA: 10^−7^, 10^−6^ M) groups. Frequency in the SAL and *E*. *coli* groups was significantly lowered by α1D-ARs antagonist and NA (three doses) compared to the CON group (Fig 7D). In the *E*. *coli* group these factors (NA: 10^−6^, 10^−5^ M) significantly raised the frequency versus the SAL group.

#### Comparison of α1-ARs isoform antagonists and NA effect to NA effect alone

Frequency in ENDO/MYO of all groups was significantly lowered by α1A-ARs antagonist and NA (10^−6^ M) (Fig 7A and 7B). α1B-ARs antagonist and NA significantly dropped this parameter in the CON (NA: 10^−5^ M) and SAL (NA: 10^−6^ M) groups, while significantly raised the frequency in the *E*. *coli* group (NA: three doses) (Fig 7A and 7C). α1D-ARs antagonist and NA significantly increased the frequency in the CON (NA: three doses) and *E*. *coli* (NA: 10^−5^ M) groups, and significantly lowered in the SAL group (NA: 10^−6^ M) (Fig 7A and 7D). α1A,B,D-ARs antagonist and NA significantly dropped the frequency in the CON and SAL groups (NA: 10–6 M) and significantly augmented it in the *E*. *coli* group (NA: 10^−5^ M) (Fig 7A and 7E).

## Discussion

The NA effect on the contractility of inflamed uterus [16, 24, 27] and ARs expression in such uteri [13] were investigated. Until now, in functional studies of ARs, participation of particular β-ARs subtypes [16] and particular α2-ARs isoforms [25] in NA effect on inflamed uterus contractility has been determined. Meanwhile, the current report is devoted to the importance of particular isoforms of α1A-ARs in contractility of inflamed uterus evoked by NA. Studies were performed on uteri with severe acute endometritis induced by intrauterine *E*. *coli* injections. In these organs inflammatory exudate was present inside the horns, and endometrium was swollen [13]. The reliability of data in the present study was confirmed by using ACh to estimate the uterine strips contractility. In healthy uteri (CON, SAL groups) ACh enhanced both the amplitude and frequency, while in inflamed uteri (*E*. *coli* group) ACh increased frequency and dropped the amplitude [29]. These data agree with our previous studies on the effect of ACh on the contractility of pig healthy uteri and those with severe acute endometritis [24, 27].

Results of NA influence on contractile response of uteri from the CON, SAL and *E*. *coli* groups were published previously [16]. Briefly, in the control and saline-injected uteri, NA led to decrease in tension, amplitude and frequency versus the period before NA addition. Above data are similar to those revealed in healthy uteri of pigs [21, 24, 30, 31] and other animal species [7, 11, 32–34]. Comparing to the period before NA application, NA in the *E*. *coli* group reduced the amplitude and frequency. In this group both parameters were lower than in the CON and SAL groups. Previous studies have shown that NA in the inflamed pig uteri reduces the amplitude and frequency [24] or increases the amplitude, but does not significantly change the frequency [27]. Moreover, here we present that in the *E*. *coli* group NA did not significantly change the tension in relation to the period before this neurotransmitter application, but the tension value was higher versus other groups.

It is worth mentioning that the present research is the first in which the participation of α1-ARs subtype and its particular isoforms in contractility of healthy pig uterus, and role of these receptors in the contractility of uterus with inflammation have been investigated. Prior to this report, investigation of α1-ARs subtype in healthy uterus had revealed their importance in an increase of human, rat and mouse myometrial contractile response [15, 35–37]. Current research showing that in uteri of the CON, SAL and *E*. *coli* groups, a greater reduction or appearance of a drop in amplitude and frequency in response to NA after blocking together A,B,D isoforms of α1-ARs is achieved compared to the action of NA alone, supports the above conclusions. Using above substances, we also found that α1-ARs subtype contributes to an increase in tension in the SAL group and the maintenance of this parameter in the *E*. *coli* group. Earlier in relation to the isoforms of α1-ARs, crucial roles of α1A-ARs in embryonic implantation [8] and of α1A-and α1D-ARs in course of late pregnancy [6, 17], as well as importance of α1B-ARs for the onset of parturition [5] in rats were reported. Application of antagonists of particular isoforms of α1-ARs (present experiment) showed that in healthy uteri, NA action increasing the amplitude and frequency is mediated by α1A- and α1D-ARs. In these uteri NA enhanced the frequency acting also by α1B-ARs. The role of α1D-ARs in NA influence on the tension was also indicated. Whereas, in the inflamed uteri, α1A-and α1D-ARs mediated in NA effect on the amplitude and frequency but not α1B-ARs. The reasons behind the lack of α1B-ARs participation in NA influence on the frequency in the *E*. *coli* group in contrast to the CON and SAL groups are unclear and need further studies. It is worth of noting that in all studied groups, compared to the NA effect alone, the appearance of a drop in myometrial amplitude and frequency (except for the SAL group) after blocking α1D-ARs and addition of the lowest dose of NA (10^−7^ M) was noted, while in response to α1A-ARs antagonist and NA at this dose above parameters did not change. These findings allow to suppose that α1D-ARs may have a greater affinity for ARs agonists (including NA) than α1A-ARs. However, this issue require further research with application of specific agonists for particular isoforms of α1-ARs. In the current study we established the partially different participation of α1A-and α1D-ARs in the contractility depending on the kind of uterine stripes. Namely, α1A-ARs participated in changes of the amplitude and frequency only in the ENDO/MYO of all study groups, while α1D-ARs played a role in NA influence in the MYO (for except the NA action on the frequency in the SAL group) and only in two cases in the ENDO/MYO (the NA action on the amplitude in the CON group and the frequency in the SAL group).

As aforementioned, in the inflammatory-changed uteri under the influence of NA, tension was increased, while the amplitude and frequency were decreased versus healthy uteri. The reduction in values of these parameters in the inflamed uteri in response to NA corresponded with the enhanced expression of β2-ARs in MYO of gilts utilized in current research [13]. Functional study showed significant role of this β-ARs subtype in a the drop of amplitude and frequency in inflamed porcine uterus [16]. In turn, the rise in tension of the inflamed uteri evoked by NA was not accompanied by increase in α1A- α1B- and α1D-ARs mRNA and/or protein expression [13]. These findings may result from the different MYO localization of particular isoforms of α1-ARs and their different ligand affinities. In rats with general inflammation the myometrial expression of mRNA α1D-ARs was increased during pregnancy, while in the nonpregnant state, the expression of this iso-form was lowered and the expression of α1B-ARs was enhanced [10]. An indirect effect of NA on the uterine contractility is also possible. Literature data show influence of NA on the synthesis/release of PGs (F2α, I2, E2) [38–40] and interaction of NA with cysteinyl LTs [41, 42]. Above PGs and LTs (C4, D4) affect contractility of the inflamed (and also healthy) uteri [24, 26–28]. It is known that PGs play a role in the increase in α1-ARs sensitivity in uterus of mature rabbits [43].

Given the clinical aspect of the importance of catecholamines, it is known that these substances released in great amounts under the effect of stressful factors exert negative action on reproductive processes i.a. through the reduction of muscular contractility in re-productive tract. Up to now it was reported that the use of non-selective β-ARs blockers (propranolol, carazolol) in cows led to improvement of uterine contractility during prevention and therapy of disorders of the post-partum period, including uterine inflammation [44, 45]. In sows, these blockers shorted the duration of labour and reduced the incidence of mastitis-metritis-agalactia syndrome [46–48]. There are no data on the use of α1-ARs drugs to modulate the uterine contractility in female animals under physiological and pathological conditions. Considering the importance of α1A-and α1D-ARs in the NA action on the particular contractile parameters of inflamed pig uterus (present experiment), we suggest that these findings may be used for developing drugs (selective agonists) increasing the contractility of inflamed uterus. They could counteract the reduction of uterine contractility in response to catecholamines and contribute to efficiency improvement of prevention and treatment of post-partum diseases (placenta retention, uterine inflammation) in animals. As a result, more favourable fertility rates would be reflected in an increase of profitability of animal production.

## Conclusions

Present study provides evidence that α1A- and α1D-ARs participate in the enhancement of amplitude and frequency of contractions caused by NA in inflamed pig uterus. Under inflammatory conditions, α1B-ARs do not mediate the effect of NA on the contractile activity of uterus. Furthermore, the involvement of α1A- and α1D-ARs in the NA action on the contractility of inflammatory-changed uterus may be used to develop agonists increasing the myometrial contractile activity during endometritis and metritis.
