# Supplementary material for: Effect of blocking of alpha1-adrenoreceptor isoforms on the noradrenaline-induced changes in contractility of inflamed pig uterus
Source: PLoS One. 2023 Feb 17;18(2):e0280152. doi: 10.1371/journal.pone.0280152 (PMC9937490; doi:10.1371/journal.pone.0280152)
Supplement: Peer review history

# Peer review: Decision Letter 0

**DOI:** 10.1371/journal.pone.0280152.r001

**Authors:** Sudarshan Kumar

21 Dec 2022

Effect of blocking of alpha1-adrenoreceptor isoforms on the noradrenaline-induced changes in contractility of inflamed pig uterus

PONE-D-22-29083

Dear Dr. Jana,

We’re pleased to inform you that your manuscript has been judged scientifically suitable for publication and will be formally accepted for publication once it meets all outstanding technical requirements.

Within one week, you’ll receive an e-mail detailing the required amendments. When these have been addressed, you’ll receive a formal acceptance letter and your manuscript will be scheduled for publication.

An invoice for payment will follow shortly after the formal acceptance. To ensure an efficient process, please log into Editorial Manager at http://www.editorialmanager.com/pone/, click the 'Update My Information' link at the top of the page, and double check that your user information is up-to-date. If you have any billing related questions, please contact our Author Billing department directly at authorbilling@plos.org.

If your institution or institutions have a press office, please notify them about your upcoming paper to help maximize its impact. If they’ll be preparing press materials, please inform our press team as soon as possible -- no later than 48 hours after receiving the formal acceptance. Your manuscript will remain under strict press embargo until 2 pm Eastern Time on the date of publication. For more information, please contact onepress@plos.org.

Kind regards,

Sudarshan Kumar, PhD

Academic Editor

PLOS ONE

Journal requirements:

When submitting your revision, we need you to address these additional requirements.

1. Please ensure that your manuscript meets PLOS ONE's style requirements, including those for file naming. The PLOS ONE style templates can be found at 

https://journals.plos.org/plosone/s/file?id=wjVg/PLOSOne_formatting_sample_main_body.pdf and 

https://journals.plos.org/plosone/s/file?id=ba62/PLOSOne_formatting_sample_title_authors_affiliations.pdf

2. Thank you for stating the following financial disclosure: 

"NO".

e) Please provide an amended Funding Statement that declares *all* the funding or sources of support received during this specific study (whether external or internal to your organization) as detailed online in our guide for authors at http://journals.plos.org/plosone/s/submit-now.  

f) Please state what role the funders took in the study.  If any authors received a salary from any of your funders, please state which authors and which funder. If the funders had no role, please state: "The funders had no role in study design, data collection and analysis, decision to publish, or preparation of the manuscript." 

Please send your amended statements by return email; we will change the online submission form on your behalf.

3. Your ethics statement should only appear in the Methods section of your manuscript. If your ethics statement is written in any section besides the Methods, please delete it from any other section. 

Additional Editor Comments:

I congratulate authors for taking up this piece of research and bringing out novel information on the various isoforms of alpha 1 adrenoreceptors active during inflammation in pig uterus under the influence of noradrenaline.

Abstract result section: Please also mention the results obtained with respect to endometrium/Myometrium

Line no. 38: “In all groups, α1A,B,D-ARs antagonist led to a greater reduction or appearance

39 of a drop of the amplitude and frequency in response to NA”. It means that there is no difference in the relative abundance of three isoforms of α1A,B,D in a healthy versus inflamed uteri. But this seems to be in contradiction with our discussion

L. No. 427-28: The reliability of data in the present study was confirmed by using ACh to estimate the uterine strips contractility”. The using Ach before and after the NA-antagonists treatment trial indicates that the strips are responding well in the experiment. However, it doesn’t mean that what was the degree of inflammation as said in the previous line “severe acute endometritis”.

Line No. 430: NA increased frequency and dropped the amplitude [29], which is in line with earlier observations.

What do you mean by earlier observations? Please mention it here for readers to understand without referring back to the references 24 and 27. In line 428 you say that Ach in healthy enhanced both amplitude and frequency while in inflamed uteri you say NA. I could not understand this difference: ACh to NA

Line no 435-440: sentences needs to be rewritten. It doesn’t explain the intent of the authors properly.

Line no, 445-449: “appearance of drop” Was it not statistically significant?

Line no. 455-56: “frequency also by alpha 1B AR”

What do you want to say you means to say that α1B-ARs only increases frequency while α1A- and α1D-ARs increases both amplitude and frequency? If so please rewrite it.

Line no 459: appearance of a reduction

Line 458-462: Please also write what do you conclude from the statement?

Line no, 462: It is possible that α1D-ARs are more sensitive to NA effect than α1A-ARs?

But antagonists have been used to block α1D-ARs so how can we conclude that it is more sensitive to NA?

Line no 464 – 467: What do authors mean by “occasionally in endo/myo”. Please be specific to conclude your findings about the participation of A and D isoforms in endo/myo or Myo.

Line no 468-469: The statement is in contrast to the statement made in line no 429.

​

# Editor report: Acceptance letter

**DOI:** 10.1371/journal.pone.0280152.r002

**Authors:** Sudarshan Kumar

9 Feb 2023

PONE-D-22-29083 

Effect of blocking of alpha1-adrenoreceptor isoforms on the noradrenaline-induced changes in contractility of inflamed pig uterus 

Dear Dr. Jana:

I'm pleased to inform you that your manuscript has been deemed suitable for publication in PLOS ONE. Congratulations! Your manuscript is now with our production department. 

If your institution or institutions have a press office, please let them know about your upcoming paper now to help maximize its impact. If they'll be preparing press materials, please inform our press team within the next 48 hours. Your manuscript will remain under strict press embargo until 2 pm Eastern Time on the date of publication. For more information please contact onepress@plos.org.

If we can help with anything else, please email us at plosone@plos.org. 

Thank you for submitting your work to PLOS ONE and supporting open access. 

Kind regards, 

PLOS ONE Editorial Office Staff

on behalf of

Dr. Sudarshan Kumar 

Academic Editor

PLOS ONE
